# Implementation of a peer support worker in a forensic psychiatric hospital in Germany—Views of patients

**DOI:** 10.3389/fpsyt.2023.1061106

**Published:** 2023-03-08

**Authors:** Peggy Walde, Julia Hadala, Verena Peipe, Birgit Angela Völlm

**Affiliations:** ^1^Department of Forensic Psychiatry, Rostock University Medical Center, Rostock, Germany; ^2^Department for Applied Psychology, SRH University Heidelberg, Heidelberg, Germany

**Keywords:** forensic psychiatry, mentally ill offender, addicted offender, peer support, recovery, implementation, qualitative research

## Abstract

**Introduction:**

Peer Support has become common in psychiatric practice in the past decade. In this article we present findings from the implementation of peer support service into a forensic mental health hospital for offenders with substance use disorders from a patient's perspective.

**Methods:**

We conducted focus groups and interviews with patients of the clinic to explore their experiences, acceptance and perceived effect of the peer support service. Data collection was conducted in two different points in time, three months and twelve months after the introduction of the of some peer support intervention. In the first time point two focus groups involving 10 patients and three semi-structured individual interviews were conducted. The second time point included one focus group with five patients and five semi-structured individual interviews. All focus groups and individual interviews were audio recorded and transcribed verbatim. Data analysis was conducted using thematic analysis.

**Results:**

Five themes emerged, (1) attitudes toward the concept of peer support work and the peer support worker himself; (2) Activities and conversation topics; (3) experiences and effects; (4) Peer support in contrast to other professions; and (5) ideas and wishes for future peer support in the clinic. In general, patients agreed on the high value of peer support work.

**Discussion:**

Findings revealed a broad acceptance of the peer support intervention by most patients, but also some reservations. They saw the peer support worker as someone who is part of the professional team, and has a unique knowledge coming from personal experience. This knowledge often facilitated conversations about several topics related to patients experiences with substance use and their recovery journey.

## 1. Introduction

Psychiatric health care systems across the world are changing. One of the most remarkable change is an orientation away from the restoration of a person's health condition to the state they had before their illness and the focus on treatment of symptoms. Instead, the term “recovery” is now seen in a broader, more holistic way. Current guidelines on the treatment of mental disorders see a recovery approach that is based on clinical symptoms only as too narrow. They state that there is a broad variance in development and outcomes where oftentimes several social, occupational and/or other impairments remain despite adequate symptom reduction (1). Despite these impairments many patients manage to develop effective strategies to regain control over their lives. Therefore, recovery is more holisticly understood as a process that highlights personal development, growth and empowerment beyond clinical symptoms. Symptom reduction steps behind leading a meaningful life and making a positive contribution to society.

A theoretical framework on recovery was developed by Leamy (2). The CHIME-framework characterizes recovery as an individual, non-linear process that is characterized by five components: (1) Connectedness, (2) Hope and optimism about the future, (3) Identity, (4) Meaning in Live, and (5) Empowerment. One important part of the first component, connectedness, is peer support. Peer support workers are individuals with lived experience of—in the case of mental health care—mental disorder who use this experience to support others in their personal recovery. In recent years this role has become more formalized and curricula for training for peer support workers have been developed (3). The concept of peer support is based on empathy and the understanding of others' experiences from a personal viewpoint, as someone with similar lived experiences of mental illness and associated struggles. This allows for an egalitarian rather than hierarchical interaction, while it promotes relationships that focus on a deeper connection that enables personal development and growth beyond traditional treatment (4). Several effects of peer support interventions have been investigated and reported in the literature. A great part of the literature so far has suggested that peer support interventions have been shown to have little effect on typical clinical outcomes like hospitalization rate and duration or symptom severity. Small positive effects have, however, been reported on feelings of hope, recovery, empowerment, and quality of life, when compared to usual treatment (5, 6). Patients described peer support workers in positive terms, e.g., providing practical and emotional support in a non-judging way. In some cases, peer support workers complemented the role of family and friends, as they made feel patients understood, even on matters that family and friends could not comprehend. The experience of someone with similar lived experiences was described as insightful and useful in everyday practice, while it also promoted hope in patients (7). More importantly, peer support work fostered a better understanding between mental health patients and mental health professionals, acting maybe as a mediator between both the different sides (8, 9).

While lately there has been a growing body of research related to peer support work and mental health, research on recovery and peer support work in forensic mental health settings is still limited. Current literature suggests that there are similarities between recovery in general mental health care and forensic mental health settings, but also some unique facets of offender recovery. Clarke et al. (10) identify six themes in their review about forensic mental health patients' views on recovery—(1) connectedness, (2) sense of self, (3) coming to terms with the past, (4) freedom, (5) hope, and (6) health and intervention. Therein, they described connectedness and sense of self as particularly important. Connectedness highlighted the importance of building and maintaining relationships with friends, family but also with hospital staff. The other theme, sense of self, was closely related to the first theme and described the interplay between self-discovery and relationships with others. The authors conclude that past relationships were oftentimes characterized with negative feelings (rejection, mistrust), whereas current and more positive relationships in the clinic might facilitate self-discovery and motivation to change. The relationship between hospital staff and patients has been suggested to be even more important in forensic settings, where contact to friends and family is limited. A negative therapeutic atmosphere and a division of “us and them” could decrease patients' engagement and motivation toward therapy. Furthermore, mistrust against staff was reported to decrease openness of patients, e.g., regarding feelings or symptoms which, in turn, lead to delayed or lack of adequate support (11). Even though, themes of connectedness and self-discovery appeared to overlap with general psychiatry, other issues are more distinctly relevant to forensic settings. For example, when it comes to recovery, forensic patients' discussed about the importance of reappraisal of one's own history and offending, and how this is linked to the recovery journey. This could lead to additional recovery barriers for forensic patients, e.g., dealing with traumatic memories, shame or being unable to forgive themselves (10). As such, and considering the impact of peer support interventions to patients, peer support work might be an effective way to address the special needs of forensic patients. Furthermore, their mediating nature might be able to close the “us and them” gap between staff and patients. Little is known about how formalized peer support work in forensic mental health settings is seen by patients, and if its effects might be similar to the one in general psychiatry settings. Introducing peer support work interventions in a secure setting might be more challenging, due to its secure nature, which might carry more risks (i.e., physical and/or psychological risks, confidentiality) or a lack of skills in the person of the peer support worker (12). Nonetheless, peer support was described as important by patients in secure settings. Shaw (12) reported on patients valuing (informal) peer support e.g., due to emotional and practical support, by offering exchanging useful information or by standing up for each other. However, since this report focused predominantly on informal peer support work, it is unclear if that could also apply to more formalized forms of peer support work. Moreover, as this report was based on settings in the UK, it would be interesting to explore how support work could be applied and perceived by patients in other health care systems.

This study aimed to explore forensic mental health patients' perspectives during the first year of the implementation of a peer support intervention. We explored the following questions:

1) What do forensic mental health patients think about peer support work in their clinic?
2) What are patients' experiences on peer support intervention, and interaction between them and the peer support worker?

## 2. Materials and methods

### 2.1. Setting

The study was conducted at the forensic-psychiatric hospital of Rostock University Medical Center in Germany. The hospital has 103 beds predominantly for individuals who have committed offenses in relation to substance use disorders (SUD). In Germany, a special paragraph of the penal code (§ 64 Strafgesetzbuch; German Penal Code) allows the treatment of individuals who were found guilty of committing serious offenses in relation to their use of licit and illicit substances, in a forensic psychiatric hospital. Their treatment is usually limited to a maximum of 2 years and independent from the individuals' criminal responsibility.

In September 2020 the forensic psychiatric clinic in Rostock hired their first peer support worker. The peer support worker was in his early 50s and had an SUD background with several SUD treatments (in general mental health settings), as well as several prison sentences served. He first became aware of the peer support training during his last SUD treatment and decided to do the training in order to “give something back” to the community. The formal peer support training was offered by EX-IN Mecklenburg-Vorpommern e.V., which is the local branch of EX-IN Germany. EX-IN stands for Experienced Involvement and is a registered charity in Germany, Austria and Switzerland. It was formed as part of the European Leonardo-Da Vinci Programme (2005–2007), a EU funded project focused on teaching and training needs of people involved in vocational and educational training (VET).

The main aim of the EX-IN e. V. is to foster the participation of people with lived experience of mental illness in their treatment process, mental health care policy making and labor market. Furthermore, the charity offers and promotes networking opportunities and has developed a curriculum for peer support worker training. This curriculum consists of five basic modules on topics like recovery, empowerment, participation and trialog (equal exchange between people with mental health experience, their relatives/carers and professionals). These modules are supplemented by seven advanced modules, e.g., regarding self-awareness, mediation, crisis intervention or teaching and learning. The basic modules comprise 110 h of training, while the advanced modules an additional 154 h. At the end of the training, which leads to a certificate, prospective peer support workers are asked to make a presentation about their development and experiences during the course (13).

The peer support worker employed in the clinic had recently received his certificate on peer support and had no previous experience of working as a peer support worker. Before the start of his post, he completed several vocational qualifications, including one in administration and one in mechanics. However, due to his SUD he was unable to continue working in these occupations and was temporarily under legal supervision. He started his position at a part time basis, 20 h per week, which later increased to 30 h per week. Professionals had generated ideas about what the peer support services in the clinic could involve, prior to the start of peer support intervention. During the first year of the intervention, the services were later adjusted and/or extended, based on personal interests and strengths of the peer support worker and according to the clinic's specific needs.

### 2.2. Overall approach

We conducted focus groups and semi-structured interviews with patients at the forensic-psychiatric hospital Rostock at two time points. Focus groups and interviews at time point 1 (T1) were conducted in the end of November and beginning of December 2020 and at time point 2 (T2) in September 2021, meaning the peer support worker was present in the clinic for 3 and 12 months respectively. More details about the participating patients can be seen in Table 1. All patients were more or less involved in the peer support intervention and had contact to the peer support worker during that time. They were asked to share their experiences in the focus groups. Individual interviews were conducted with patients who had had more intensive contact with the peer support worker. Individual interviews were thought to be a able to provide a better insight into the topic, while focus groups to provide a wider perspective. For a more holistic view we also conducted focus groups with staff. The results are not described here.

**Table 1:** Characteristics of participants in focus groups and interviews at time point T1 and T2.

		**T1: November/December 2020**	**T2: September 2021**
Focus group	N	10 (♀= 2)	5 (♀= 0)
	Age	19-47 yrs (mean: 30.6 yrs)	32-48 yrs (mean: 39.0 yrs)
	Primary Diagnosis		
	- SUD	8	4
	- Other	2	1
Semi-structured interviews	N	3 (♀= 0)	5 (♀= 0)
	Age	32-47 yrs (mean: 37.0 yrs)	23-43 yrs (mean: 31.8 yrs)
	Primary Diagnosis		
	- SUD	3	4
	- Other	0	1

There were two focus groups conducted in T1 with 4 and 6 participants each and one focus group at T2. SUD Substance Use Disorder, Other comprises primary diagnosis of personality disorder or schizophrenia

### 2.3. Sampling and recruitment

Our sample included patients of the forensic psychiatric clinic in Rostock University Medical Center, who had been involved in the peer support intervention, e.g., in group therapeutic settings, PSW-led activities, such as baking or the recovery group led by the peer support worker, or in one-to-one sessions. Patients who were perceived as lacking capacity to provide an informed consent and patients under the age of 16 years were excluded. Except of one person, all patients were German native speakers.

Patients were invited to participate by one of the authors (PW). The author visited the clinic's wards at a time when most of the patients were together in the living room (e.g., morning rounds or the beginning of therapeutic group sessions). Patients were informed about the research project, the main aims and objectives, and were invited to participate. Patients had the opportunity to ask any questions they might had. Those who were interested were given a written participant information sheet and a consent form to fill in case they decided to take part. Information about their capacity to give informed consent was given to the researcher by the therapeutic team.

Table 1 shows the characteristics of participating patients according to time point. We only collected characteristics that were relevant for answering the questions.

### 2.4. Data collection

Focus groups and interviews were arranged in the clinic and followed an interview guide developed by two of the authors (PW, BV). Patients were asked about their experiences with the peer support worker, their conversation topics, if they recognize any effects due to his presence and their ideas and wishes for the future development of peer support within the clinic. All data were collected face-to-face by one of the authors (PW) who has a Master's degree in psychology, while a research intern assisted in the focus groups. The focus groups' length varied between 43 to 86 min (with an average of 66 min), while the interviews' duration was between 16 and 50 min (with an average of 33 min). All focus groups and interviews were conducted in German, were audio recorded and transcribed verbatim. Data were anonymized before analysis. Any personal information, including names, or any information that could lead to the identification of a person (e.g., names, places) were removed or revised accordingly (e.g., “fellow patient” instead of a name). All patients were given IDs (e.g., P1) to ensure confidentiality.

### 2.5. Data analysis and trustworthiness

The pseudoymized transcripts were analyzed in NVivo 12 pro. We used Thematic Analysis (14) to identify patterns across data sets. Focus groups and semi-structured interviews were analyzed separately from each other and by time point to discover the salient themes within the different time points. We used an inductive approach at a semantic level. Three of the authors were involved in the coding (PW, HJ, VP). A third of all interviews and focus groups were double coded. Here, the initial coding was done by each author first by themselves. After the initial coding was completed, the authors came together, compared and discussed their codes and themes and agreed on a joint coding scheme. This scheme was discussed with the fourth author (BV), who was not involved in the coding process so far but supervised the overall project. The following transcripts were integrated into that scheme and revisions were made, where necessary (e.g., when new themes emerged). We originally intended to explore the development of the patient's attitudes toward the peer support concept and the peer support worker as a person within the first year after the concept implementation. As analyses collected in the 3rd and 12th month after the start of the peer support intervention revealed similar findings, the authors decided to combine analyses into one. Therefore, what we present here is the results across all data sets. Interview quotations were translated as close as possible to the original text to reflect patient's views.

### 2.6. Ethical approval

Ethical approval was granted by the Rostock University Medical Centers Committee of Ethics. All participants were informed about the purpose of the study and had sufficient opportunities to ask questions before giving informed consent.

## 3. Results

During the interviews and focus groups, several themes emerged. Herein we focused on shared themes that occurred during time point T1 and T2 in individual interviews as well as focus groups. Main themes were: (1). patients' attitudes toward the peer support worker, including reservations about the concept or the actual person, (2) the activities (including conversation topics) the patients had with the peer support worker, (3) the experiences and effects the patients reported about their interaction with the peer support worker, (4) contrasts of the peer support worker with other professions, and (5) ideas and wishes for the future of peer support work in the clinic. All themes are presented in Table 2.

**Table 2:** Shared main themes and subthemes related to peer support work in the clinic that emerged during interviews and focus groups.

**Themes**	**Sub-Themes**
Attitudes towards the concept of peer support work and the peer support worker himself	Positive attitudes
	Initial reservations
Activities and conversation topics	-
Experiences and effects	Easier connection through similar experiences
	Effects on patient's attitudes Hope and motivation Unpleasant experiences
Peer support in contrast to other professions	-
Ideas and wishes for future peer support work	For the current peer support worker
	For additional, future peer support workers

### 3.1. Attitudes toward the concept of peer support work and the peer support worker himself

This theme incorporates patients' thoughts and feelings about peer support as a concept, and in particular thoughts and feelings about the peer support worker employed in the clinic. None of the interviewed patients had been familiar with the concept of peer support work before the implementation of this intervention in the clinic. Patients both in the focus groups and in individual interviews were found to have a *positive attitude* toward the concept of peer support work after getting in contact with it. According to them, most fellow patients (some who were not involved in the study) were positive toward peer support. Patients mentioned that a peer support worker, due to their personal experience, they are in a better position to understand what they are going through, in comparison to other members of mental health care staff.

“*And yes, I find it actually really good that there is something like that [peer support work]. It is actually something different to talk with someone who made these experience himself than, well, hearing that from a therapist who, you know, has their knowledge from textbooks and studies and so on, but has never made the experience themselves and doesn't know how it is to be dependent and addicted.” (EinzelPat1_T1)*

There is unanimity of this belief, as statements like this were found in all focus groups and interviews. Patients explained the similarity between peer support worker's experiences and their own experiences with substance abuse, related issues and related offending, created a common ground, which opened a space for discussion and promoted feelings of deeper understanding and connection. This altered the way patients approach disclosure and sharing, in such settings and led to more openness. As such, patients appreciated the presence of a peer support worker as an additional member of staff. Patients also made references to specific personal characteristics of the peer support worker, which they appreciated and felt that had an impact on his work, being sympathetic, open, and honest. Patients expressed respect for this recovery history with which he was potentially seen as a role model for the individuals.

“*It is crazy that a man who was addicted to drugs, heavily addicted, can suddenly lead a very normal life. I would never had never thought that.” (EinzelPat3_T2)*

Some patients had some *initial reservations toward the peer support worker*; some talked about reservations they had themselves or reservations expressed by other fellow patients. It became clear that, because patients were not familiar with the concept of (formalized) peer support, some were unsure about the main purpose of peer support or what a peer support worker could be able to add to the work been done in the clinic. They expressed a general mistrust in some fellow patients and a fear to be sounded out. This fear could be attributed to various factors, such as first the general unfamiliarity with the concept of peer support, second, their long history of socialization within institutions and with criminal peers that might make them more reluctant to trust new people and new members of staff. Insecure behavior of the new peer support worker, when he started his work on the ward, was also noted, and could be another contributing factor. This behavior was described as “flapping around” and having some issues in getting in contact with patients. One patient in a focus group summarized his experiences:

“*[…] because many patients don't know that, this peers support thing, it is initially biased, so that you are suspicious. I mean, I also noticed that with me in the group, that some of them [the fellow patients] asked ‘Why are you going to him? What are you doing there?' and so on. Just mistrust, well, mistrust. Only seeing the bad.” (FoGruPat_T1)*

To some extent, this rejection might have been the result of mutual insecurity. On the other hand, patients also reported that some of their fellow patients tended to reject any therapeutic offers, including the peer support worker. In general, patients who rejected the peer support worker were described to be a minority and the patients who participated in the study attributed most of these reactions to a lack of chemistry, between the peer support worker and other patients, and not to behavior and skills of the peer support worker.

### 3.2. Activities and conversation topics

This theme describes the various activities included in the peer support intervention, as well as topics that came up during activities, as experienced and described by patients.

Patients explained that the peer support worker played the role of the mediator between patients and other members of staff. One patient described it as follows:

“*But he works with us but also with the nurses, doctors, at least with the psychologists. Exchange of his experience to educate them, what they don't understand and supporting us, to join us on our journey […] but at the same time supports the instruments of justice [meaning the clinic staff], to explain things a little better because finally the[ir] reports are sent to the Justice. To make sure they are correct[…]” FoGruPat_T2*

Patients also talked about other everyday activities that the peer support worker was involved, like cooking, or be present at ward rounds and building rapport with the patients. All of the patients mentioned that they had the opportunity to have one-to-one conversations with the peer support worker. During these conversations, but also in the informal ward meetings several topics for discussion came up, e.g., related to the past of the peer support worker, his recovery journey and his experiences with offending and imprisonment, due to his substance abuse. Patients in mentioned that some people had sought advice and practical tips from the peer support worker, e.g., regarding managing craving, substance use, relapse or about opiate substitution therapy or for the time after discharge from the institution. Other topics included discussing current events taking place in the clinic or daily issues on the ward.

It became obvious that the peer support worker covered several roles for patients. In the occasions that the peer support worker acted as a mediator, between them and other members of staff, patients felt that he advocates for them, they trusted his words and felt that he has always been acting (and speaking) on their best interests. This trust was built not only in one-to-one conversations, but also in informal, occasional chats during everyday activities, like cooking or baking or during visits on the wards. It was also noted that trust was established by listening the peer support worker talking to other fellow patients.

### 3.3. Experiences and effects

In this theme patients described their interaction with the peer support worker and how this has influenced them, e.g., in their way of thinking or behaving. Patients described that it was easier for them to find a connection to the peer support worker (*Easier connection through similar experiences*), compared to other professions, due to their shared experiences with drugs, substance use and the offenses they committed in relation to that.

“*It is different to talk about that, also easier to talk. Just experience, he has the experience and someone else, who has no such experience, there it is pointless to talk. They want to help, sure, but they cannot feel what we felt at that time [when they were still consuming substances]. At least I think so.”(EinzelPatT1_3)*

This shared experiences, but also the personality of the peer support worker himself made it easier for some patients to trust him. This helped some of the patients to rethink their attitudes (*Effects on patient's attitudes*). In the one-on-one interviews, the patients described an altered view on therapy and addiction. They decided to open up in therapy and speak openly about their experiences, e.g., their feelings of craving and other issues, in group therapy sessions. Another patient decided to start reporting drug-related activities amongst patients on the ward to staff, which he had not done before.

“*He took the time to talk about that with me, so that in the end, I was like ‘Yes, he is right. I can agree with that, can answer for that to myself' and now when I report this to the clinic [that there are drugs on the ward] or whatever, when I tell that, I have no longer a bad conscience, that I would be a grass, you know. Then I see it as having responsibility, you know, for my children, family and so on and I do this to protect myself and it has nothing to do with grassing. That was a really important experience. I always struggled with that, it always stood between me and my therapy if you cannot distance yourself from that in order to not be a grass” (EinzelPatT1_3)*

Furthermore, peer support worker's personal experience with substance abuse and therapy made him a good advisor for patients. Patients felt that he was in the position to provide information on queries related to emotional and practical issues that other members of staff could not. More particularly, the peer support worker was able to provide information about strategies for dealing with cravings and on how to stay sober in difficult situations. Patients appreciated advice and knowledge exchange on such useful for them topics.

“*In the end, the conversation with the peer support worker was a real help. Yeah, he could put himself into my shoes better and then told me, what he did, how he did it. So one could use his experience, that he had, you know, and try to somehow apply them. Well, I don't know, the other [members of staff] learned at university about craving and such, they say it comes and goes and we had to bear it for half an hour. Yeah, but what you can really do or how one can calm oneself down is something no nurse can tell you. And with that, only the peer support worker really helped me*.” *(FoGruPat_T1)*

Finally, the peer support worker was able to instill hope in patients and motivate and empower them (*Hope and motivation*). Patients reported the peer support worker had encouraged them to engage in their therapy and to ask for help. Other patients have mentioned that peer support intervention has helped them in starting developing plans for their future.

There was also a small minority of patients, who discussed adverse experiences with the peer support worker (*Unpleasant experiences*). These experiences came up unintendedly by something the peer support worker said or did. One patient gave an example in which he felt triggered by the peer support worker's use of common plastic bags to transport some cooking ingredients:

“*…and there was one thing that bothered me when we cooked together for the first time. He brought his herbs from his garden, which symbolized something, the symbol was how he packed them […] that was unpleasant for me. He packed that in little packets like the ones [a dealer] used to sell dope.”* (FoGruPat_T2)

Examples like this were very rare in the focus groups and interviews. In general, patients described positive interactions and experiences. Having shared experiences seemed to acted as a “door opener” that made patients more open to discussion, arguments and more willing to accept other points of view. Furthermore, the peer support workers feedback seems to foster patients self-reflection and might enable them to recognize self-deception. Nonetheless, peer support work also seemed to come with some adverse events. It appears possible that a long history of substance use and related criminal behavior might lead to internalized behaviors that the peer support worker was not aware of. These behaviors would trigger bad feelings, e.g., emotional stress or craving, in some patients.

### 3.4. Peer support in contrast to other professions

This theme was about differences between the peer support work profession and other professions. Patients described they felt better understood in some topics by the peer support worker than by other professions, e.g., therapists or nurses. Due to shared experiences, the peer support worker was able to relate to patients experiences such as cravings or certain drug use or prison related behavior, in a way that was difficult for other professionals in the clinic. As one patient stated:

“*And when I sit there with the psychologist and tell her something from prison, there is little understanding, or credibility, so to say.” (FoGruPat_T1)*

This increased credibility attached to the peer support worker seemed to make it easier for patients to accept input and advice from him, in comparison to other members of staff. Nonetheless, patients see the peer support worker as a supplement to the usual therapy and not as substitute and acknowledged that each side, such as psychologists, psychiatrists, nurses and peer support workers, had an important and different contribution to the therapeutic process. Within this therapeutic setting, patients felt that peer support work could not replace therapy, but might help to support patients to gain trust in the therapeutic team, and to encourage openness in therapy.

### 3.5. Ideas and wishes for future peer support work

In this theme involves patients' ideas for the development of peer support work in the clinic, based on their current experiences. All patients involved in the study stated that they wished the peer support intervention in the clinic to be continued. *For the current peer support worker*, they thought that it would be beneficial for them if he was more actively and regularly involved in group therapy sessions offered in the wards. It should be noted here that at the time of these study, the peer support worker was only involved in the group therapy sessions in the ward where he was formally assigned to and visited other wards' group therapy sessions only upon special requests. Patients also suggested that regular ward-internal group sessions led by the peer support worker, with patient participation on a voluntary basis and without presence of other staff could be another activity involved in peer support intervention. An example of this might have been the recovery group that was conceptualized and led by the peer support worker on the admissions ward. This group covered several recovery relevant topics, but was also used these meetings as space to discuss current issues and topics affecting the patients. Some patients also mentioned they would appreciate the presence of the peer support worker on other special occasions, e.g., during alcohol exposition or when they are on leave outside the clinic, e.g., looking for accommodation for the time after discharge.

Patients also thought that an additional peer support worker might be a good idea for the future. They gave several reasons for that. First, patients thought that it might be too much work for one person, offering peer support to the whole population of the clinic. One or more additional peer support worker(s) would be able to address the desire for more participation of peer support worker(s) in other activities, such as group therapy sessions, while it would also make it possible for the peer support worker(s) to have a stronger presence in the wards and establish rapport with the patients. Furthermore, having another peer support worker in the team might resolve issues attributed to a lack of sympathy between patient and a special peer support person as well as issues attributed to personal characteristics, such as gender, age, ethnic background. Some patients, for instance, might find hard to connect with a peer support worker of the opposite gender, which is something that could be resolved with the addition of another peer support worker. Ideas about characteristics *for an additional, future peer support worker* covered topics of gender or diagnostic background. Patients stated that they could imagine a female peer support worker in the clinic (a) supporting the female ward with their special needs and, (b) bringing in a female perspective to the male patients, especially since many of them do have histories of complicated relationships with women. Moreover, patients suggested that a peer support worker who has other lived experiences, such as more experiences with other types of substances, like new chemical drugs, or a diagnosis of a personality disorder could also be an effective addition to the team. This is important, especially considering the high prevalence of dual diagnosis or co-morbidity of mental health disorders in forensic mental health settings. It was finally noted that a criminal past would be necessary to understand the subtle signs and intentions of patient's communication amongst each other. Just a diagnosis of a substance use disorder was seen as insufficient for this setting.

## 4. Discussion

The present study explored forensic mental health patients' experiences and perceptions of a newly implemented forensic peer support intervention. Patients were found to be unfamiliar with the concept of peer support, but had expressed unanimous support over it after the start of the intervention. Patients described increased feelings of connectedness with the peer support worker and felt being better understood by someone who has the same or similar experiences with them. They felt empowered by the peer support worker to engage in their own therapy and they were happy to discuss ideas for the future of peer support interventions within the clinic. According to our results, peer support was not seen as competitor to other professionals, but as an additional member of the team that could make an important contribution (to the professional team and individuals' therapy), such as support the engagement of patients to therapy. There were a few cases that patients rejected the peer support worker. It has been concluded that this could be probably attributed to lack of connectedness with the peer support worker, due to personal characteristics, reasons and/or personal aversion; something that could potentially be resolved with the addition of more peer support workers, possessing different characteristics. Our results point at two directions: first, in cases where peer support intervention is newly implemented, and many patients lack familiarity with the concept of peer support, it might be useful to consider including multiple preparation sessions for the peer support worker prior to the start of the intervention. And second, the acceptance of a new peer support worker is not a natural course of action. The reaction of patients toward the peer support worker and the interaction amongst them has to be carefully observed and evaluated at the early stages, in order to prevent undesirable developments, and maybe even to protect the peer support worker from hostile patient behavior.

Many findings from our focus groups and interviews matched what other scholars have suggested about peer support work in general psychiatry, e.g., that patients felt understood and accepted and that the peer support workers are able to instill hope (5, 6, 15, 16). The reports of some patients about reconsidering therapy (and engaging more with it), start reporting drug-related activities in the clinic and begin making plans for their lives after discharge, can be seen as steps toward taking responsibility for themselves. We saw these reports as an indication of patient empowerment, which was another commonly reported effect of peer support work in general psychiatry (6, 15).

In addition to these points, patients raised some other issues that might be more setting specific. They expressed some reservations they had with regards to the presence of the peer support worker in the wards, while they talked about the usefulness of a having a peer support worker in a secure hospital. According to the literature, general psychiatry patients' did not express such skepticism. Therefore, it might be possible that an increased skepticism is more relevant to forensic mental health settings, and that there might be a need of special measures to deal with it, such as extensive training and preparation of prospective peer support workers in such settings. Furthermore, patients suggested that having a criminal past is essential for a peer support worker in a forensic setting. Without having such a background, a peer support worker might not be in the position to recognize or might misinterpret the subtle elements of forensic patients' communication, intentions and behavior. Moreover, issues in handling memories and feelings about the own offense were described in forensic populations (10). The exchange with a peer support worker with similar experiences could be beneficial for these patients, too. Even if the offending history seemed to play a minor role in the conversations between patients and peer support worker, it represents a connecting element between them that enhances the feeling of connectedness. This feeling can be important for patients' individual recovery process. Therefore, hiring a peer support worker with an offending history seems an important consideration to make, when designing a peer support intervention for a forensic settings. Moreover, since the contact to family and friends is limited, in such settings, a peer support worker might be an important source of feeling connected (within the clinic and to the outside world) and understood. Because of that, but also because of improving the understanding between patients and clinical staff peer support might be able to bridge the “us and them” gap that was often described by patients (11). Some initial thoughts on the impact of the peer support intervention included patients starting to open up to therapy and to report drug related incidents to staff. In doing so, patients also made the first step in getting appropriate support for their individual needs.

Despite the positive points raised, there were a few patients who reported having (observed) issues in building trust with the peer support worker and rejected his support. A general, often occurring mistrust against staff was described in the literature (11), but, to our knowledge, has not been previously suggested to apply to (forensic) peer support workers. Peer support workers, who are new to this field of work, should receive adequate support and training prior to their start in the role and supervision should be taken seriously. Another interesting point that this study raised, is that some patients were unintendedly triggered by things the peer support worker said or did. Some actions that might look normal to people without criminal background, could be interpreted by forensic patients in another way and trigger certain thoughts and feelings e.g., feelings of cravings. It is difficult to tell if this is something only relevant to peer support workers (be in the position to trigger certain feelings or thoughts), or if this something that could be caused by other members of staff or peer support workers in other settings too. Furthermore, we discussed the possibility that some behaviors from a criminal past became internalized and are not recognized as such from the peer support worker but by patients (e.g., the way cooking ingredients are wrapped or sharing goods). To be aware of that, good communication and feedback from colleagues as well as competent supervision appears to be important for de-escalation and professional reasons.

The results showed that peer support work was widely accepted and appreciated by most patients in a forensic mental health setting. Patients explained that for them it was important that the peer support worker had experience of both incarceration and substance abuse, as that made him more credible as a person and his work more effective. From the discussions in focus groups and individual interviews, it became clear that patients, who saw various parallels between their history and the history of the peer support worker were more likely to contact and approach the peer support worker (or be more open to be contacted and approached by him). These results match the findings in literature, in which shared experiences are essential for successful peer support work. Therefore, we recommend forensic institutions who plan the implementation of peer support to consider these points and be open to applicants with a criminal history.

### 4.1. Study limitations

Some study limitations should be mentioned. First, the setting in which the peer support intervention was implemented was quite special. In contrast to other countries practices, Germany offers the opportunity to people who have committed an offense, related to substance abuse disorder, to be placed in a forensic mental health institution instead of prison. Since our sample consisted predominantly of individuals who have committed an offense related to substance use disorder and/or with people with dual diagnosis of substance abuse disorder and personality disorder, our results might not account for forensic mental health patients with other conditions, such as schizophrenia or an affective disorder background. Therefore, we recommend further research to cover the special needs of patients with different conditions. This, together with the qualitative nature of the study might limit study's generalizability to other contexts. Furthermore, the process of patient recruitment encouraged self-selection, in a way that only patients with a positive attitude toward peer support work might have participated. Therefore, possible critical voices might have been missed or underrepresented. Also minority groups might be underrepresented, such as female patients or patients whose native language is not German. The latter patient group might be more reluctant to participate, especially in focus groups, because of language barriers. Therefore, further research could focus on special needs and requirements these subgroups might have regarding peer support work.

## Data availability statement

The raw data supporting the conclusions of this article will be made available by the authors, without undue reservation.

## Ethics statement

The studies involving human participants were reviewed and approved by Ethikkommission an der Universitätsmedizin Rostock (Committee of Ethics at Rostock University Medical Center). The patients/participants provided their written informed consent to participate in this study.

## Author contributions

PW and BV contributed to the conception and design of the study. PW, JH, and VP performed the qualitative analysis which was supervised by BV. PW wrote the first draft of the manuscript. BV contributed to manuscript revision. All authors read and approved the submitted version.
